# Molecular Epidemiology of Mycobacterium abscessus Isolates Recovered from German Cystic Fibrosis Patients

**DOI:** 10.1128/spectrum.01714-22

**Published:** 2022-08-08

**Authors:** Nils Wetzstein, Margo Diricks, Thomas A. Kohl, Thomas A. Wichelhaus, Sönke Andres, Laura Paulowski, Carsten Schwarz, Astrid Lewin, Jan Kehrmann, Barbara C. Kahl, Karl Dichtl, Christian Hügel, Olaf Eickmeier, Christina Smaczny, Annika Schmidt, Stefan Zimmermann, Lutz Nährlich, Sylvia Hafkemeyer, Stefan Niemann, Florian P. Maurer, Michael Hogardt

**Affiliations:** a Department of Internal Medicine, Infectious Diseases, University Hospital Frankfurt, Goethe University, Frankfurt am Main, Germany; b German Center for Infection Research (DZIF), partner site Hamburg-Lübeck-Borstel-Riems, Hamburg, Germany; c Molecular and Experimental Mycobacteriology, Research Center Borstel, Borstel, Germany; d Institute of Medical Microbiology and Infection Control, University Hospital Frankfurt, Goethe University, Frankfurt am Main, Germany; e National and WHO Supranational Reference Laboratory for Mycobacteria, Research Center Borstel, Leibniz Lung Center, Borstel, Germany; f Division of Cystic Fibrosis, Department of Pediatric Respiratory Medicine, Immunology and Critical Care Medicine, Charité-Universitätsmedizin Berlin, Berlin, Germany; g Division of Cystic Fibrosis, CF Center Westbrandenburg, Campus Potsdam, Klinikum Potsdam, Potsdam, Germany; h Unit Mycotic and Parasitic Agents and Mycobacteria, Robert Koch Institute, Berlin, Germany; i Institute of Medical Microbiology, University Hospital Essen, University Duisburg-Essen, Essen, Germany; j Institute of Medical Microbiology, University Hospital Münster, Münster, Germany; k Max von Pettenkofer Institut, Institute of Medical Microbiology and Hygiene, Medizinische Fakultät, Ludwig-Maximilians-Universität, Munich, Germany; l Department of Respiratory Medicine and Allergology, University Hospital Frankfurt, Goethe University, Frankfurt am Main, Germany; m Christiane Herzog CF Center, Medical Clinic, Department of Respiratory Medicine and Allergology, University Hospital Frankfurt, Goethe University, Frankfurt am Main, Germany; n Division for Allergy, Pneumology and Cystic Fibrosis, Department for Children and Adolescence, University Hospital Frankfurt, Goethe University, Frankfurt am Main, Germany; o Interfaculty Institute of Microbiology and Infection Medicine Tübingen, Insitute for Medical Microbiology and Hygiene, University Hospital Tübingen, Tübingen, Germany; p Department of Infectious Diseases, Medical Microbiology and Hygiene, Heidelberg University Hospital, Heidelberg, Germany; q Department of Pediatrics, Justus-Liebig-University Giessen, Giessen, Germany; r Mukoviszidose Institut, gemeinnützige Gesellschaft für Forschung und Therapieentwicklung mbH, Bonn, Germany; s Institute of Medical Microbiology, Virology and Hospital Hygiene, University Medical Center Hamburg-Eppendorf, Hamburg, Germany; t German National Consiliary Laboratory on Cystic Fibrosis Bacteriology, Frankfurt am Main, Germany; University Paris-Saclay, AP-HP Hôpital Antoine Béclère, Service de Microbiologie, Institute for Integrative Biology of the Cell (I2BC), CEA, CNRS

**Keywords:** *Mycobacterium abscessus*, cystic fibrosis, whole-genome sequencing, dominant circulating clones, hospital transmission, German CF registry, nontuberculous mycobacteria

## Abstract

Infections due to Mycobacterium abscessus are a major cause of mortality and morbidity in cystic fibrosis (CF) patients. Furthermore, M. abscessus has been suspected to be involved in person-to-person transmissions. In 2016, dominant global clonal complexes (DCCs) that occur worldwide among CF patients have been described. To elucidate the epidemiological situation of M. abscessus among CF patients in Germany and to put these data into a global context, we performed whole-genome sequencing of a set of 154 M. abscessus isolates from 123 German patients treated in 14 CF centers. We used MTBseq pipeline to identify clusters of closely related isolates and correlate those with global findings. Genotypic drug susceptibility for macrolides and aminoglycosides was assessed by characterization of the *erm*(41), *rrl,* and *rrs* genes. By this approach, we could identify representatives of all major DCCs (Absc 1, Absc 2, and Mass 1) in our cohort. Intrapersonal isolates showed higher genetic relatedness than interpersonal isolates (median 3 SNPs versus 16 SNPs; *P* < 0.001). We further identified four clusters with German patients from same centers clustering with less than 25 SNPs distance (range 3 to 18 SNPs) but did not find any hint for in-hospital person-to-person transmission. This is the largest study investigating phylogenetic relations of M. abscessus isolates in Germany. We identified representatives of all reported DCCs but evidence for nosocomial transmission remained inconclusive. Thus, the occurrence of genetically closely related isolates of M. abscessus has to be interpreted with care, as a direct interhuman transmission cannot be directly deduced.

**IMPORTANCE** Mycobacterium abscessus is a major respiratory pathogen in cystic fibrosis (CF) patients. Recently it has been shown that dominant global clonal complexes (DCCs) have spread worldwide among CF patients. This study investigated the epidemiological situation of M. abscessus among CF patients in Germany by performing whole-genome sequencing (WGS) of a set of 154 M. abscessus from 123 German patients treated in 14 CF centers. This is the largest study investigating the phylogenetic relationship of M. abscessus CF isolates in Germany.

## INTRODUCTION

Mycobacterium abscessus is a multidrug-resistant rapid growing nontuberculous mycobacterium (NTM) causing lung infections in predisposed individuals or soft tissue infections after surgical procedures (1, 2). It is a major cause of morbidity and mortality in patients with predisposing lung diseases, such as cystic fibrosis (CF) or non-CF-bronchiectasis (3). In CF patients, infection with M. abscessus leads to an accelerated decline in lung function (4, 5) and is considered a contraindication for lung transplantation in most CF-centers (6–8). Over the last 2 decades, increasing prevalence rates for NTM lung infection in CF patients have been reported. However, they vary widely among countries and between different centers, currently ranging from 0% to 7.2% in European countries (in adults from 0% to 11.1%) (9). M. abscessus is the predominant species in the European CF population (9). In contrast, the majority of pulmonary NTM infections in people with CF in the US are caused by M. avium complex (MAC) (10).

As M. abscessus is inherently drug resistant to most anti-infective agents, infections are extremely hard to treat and eradication remains often unsuccessful. Treatment success rates for M. abscessus pulmonary disease have been shown to be only 25% to 58% (5, 11). Current treatment guidelines recommend a prolonged and intense combination therapy consisting of several antibiotic agents with significant adverse effects (12, 13), with macrolides and aminoglycosides (intravenously or inhalative) being the backbones of the therapy (12–14).

M. abscessus comprises three subspecies, M. abscessus subsp. *abscessus* (*mabs*), M. abscessus subsp. *massiliense* (*mmas*), and M. abscessus subsp. *bolletii* (*mbol*) (15). This taxonomic distinction is clinically relevant as most *mabs* and *mbol* isolates show inducible resistance to macrolides due to a functional *erm*(41) gene that encodes an erythromycin ribosome methyltransferase. In *mmas*, this gene is truncated, rendering this subspecies generally susceptible to macrolides, which is associated with better treatment response and clinical outcome (16). Constitutive macrolide resistance in all three subspecies can be conferred via mutations in the *rrl* gene (A2058C, A2058G, A2058T, A2059C, A2059G, and A2059T Escherichia coli numbering) encoding 23S rRNA (17), whereas mutations in the *rrs* gene (A1408G, T1406A, and C1409T) encoding 16S rRNA can lead to aminoglycoside resistance (18).

In recent years, so-called dominant circulating clones (DCCs) have been described among CF patients suggesting a global spread and the possibility of human-to-human transmission (direct and indirect) (19, 20). Moreover, representatives of the DCCs have been shown to be more virulent *in vivo* and *in vitro* (20, 21). Recently, it has been hypothesized that niche adaptation of M. abscessus in smokers preceded its spread in the CF community in the 1960s (22). Until now, DCCs have been described in CF-patients from different countries (23, 24), and interestingly in non-CF-patients, as well (25). In a prior single center analysis, we demonstrated that they are also present in Germany, but comprehensive data from CF patients in Germany is missing so far (26).

The aim of this work was to evaluate the prevalence and species distribution of NTM among CF patients in Germany, as well as testing frequency, using data from the German CF patient registry (27). In addition, we aimed to investigate whether the DCCs are present in a comprehensive set of M. abscessus isolates from German CF patients and to identify possible transmission clusters using a whole-genome sequencing approach.

## RESULTS

### NTM epidemiology in German CF patients.

**FIG 1:**
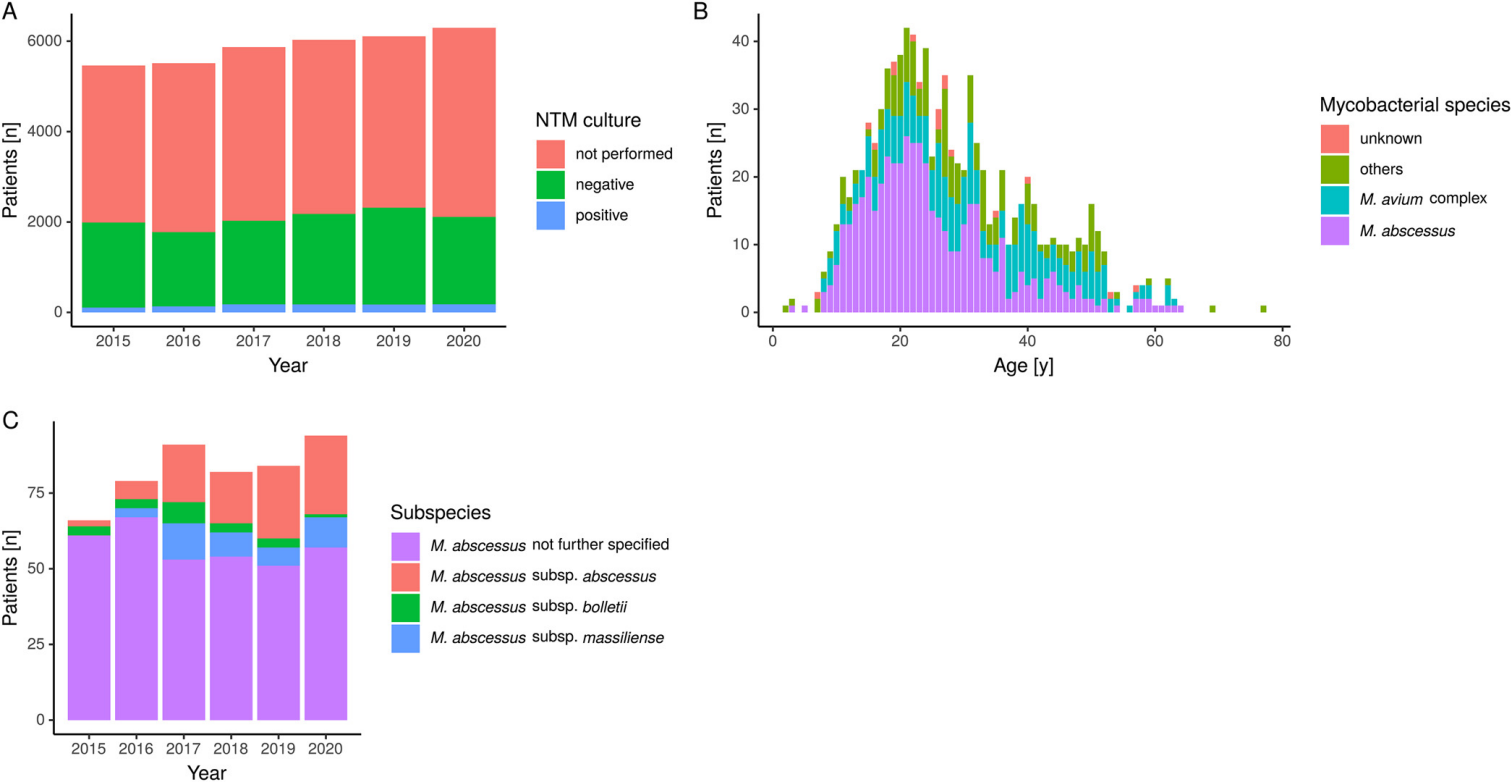
(A) Total number of tested CF-patients, negative and positive NTM results from 2015 to 2020 as reported in the German CF registry. (B) Species and age distribution of patients with positive NTM cultures (raw data are not shown). (C) Subspecies distribution for M. abscessus as reported in the German CF registry, 2015 to 2020.

From 2015 to 2020, the number of patients documented in the CF registry increased from 5,462 patients to 6,295 patients (Table 1). Of those, between 32.2% and 37.9% were tested for NTM and 1.9% to 3.0% had positive test results (Table 1, Fig. 1A). The most frequently isolated mycobacterial species was M. abscessus accounting for 46.6% to 62.3% of cultured isolates per year, followed by MAC (15.1% to 36.3%) and others (19.6% to 23.7%, including M. kansasii, M. fortuitum, M. gordonae and M. chelonae). Among children, M. abscessus is the leading NTM in Germany (45.9% to 78.1%), whereas MAC was cultured in 9.4% to 36.1% (Fig. 1B). In adults, other NTM such as MAC become more important (17.6% to 36.4%), but M. abscessus remains predominant (40.3% to 56.1%). For the majority of M. abscessus isolates, the subspecies was not reported to the registry (69.2%). Of those specified (*n* = 153), *mabs* was the most frequent one with 61.4% during the 6-year period (Table 1, Fig. 1C).

**TABLE 1:** Summarized NTM data from the German CF registry 2015–2020

Patient characteristics	2015	2016	2017	2018	2019	2020
Patients in registry^*a*^	5,462	5,512	5,869	6,031	6,108	6,295
Patients tested for NTM	1,989 (36.4^*e*^)	1,775 (32.2^*b*^)	2,026 (34.5^*b*^)	2,178 (36.1^*b*^)	2,316 (37.9^*b*^)	2,112 (33.6^*b*^)
Patients with positive culture^*e*^	106 (1.9^*b*^;5.3^*c*^)	133 (2.4^*b*^;7.5^*c*^)	177 (3.0^*b*^;8.7^*c*^)	176 (2.9^*b*^; 8.1^*c*^)	174 (2.9^*b*^;7.5^*c*^)	179 (2.8^*b*^;8.5^*b*^)
M. abscessus	66 (62.3^*d*^)	79 (59.4^*d*^)	91 (51.4^*d*^)	82 (46.6^*d*^)	84 (47.5^*d*^)	94 (52.5^*d*^)
*mabs*	2	6	19	17	24	26
*mmas*	0	3	12	8	6	10
*mbol*	3	3	7	3	3	1
Subspecies not known	61	67	53	54	51	57
MAC	16 (15.1^*d*^)	38 (28.6^*d*^)	48 (27.1^*d*^)	55 (31.3^*d*^)	51 (28.8^*d*^)	65 (36.3^*d*^)
Others	22 (20.8^*d*^)	14 (10.5^*d*^)	37 (20.9^*d*^)	39 (22.2^*d*^)	42 (23.7^*d*^)	35 (19.6^*d*^)
M. kansasii	2	1	2	4	2	0
M. fortuitum	2	0	3	0	6	3
M. gordonae	1	4	15	15	17	13
M. chelonae	2	3	4	4	7	9
Miscellaneous	15	6	13	16	10	10
Unknown	2 (1.9^*d*^)	4 (3.0^*d*^)	4 (2.3^*d*^)	5 (2.8^*d*^)	1 (0.6^*d*^)	1 (0.6^*d*^)

a In each year, patients who had a prior transplant were excluded.

b Percentage of all patients in registry.

c Percentage of tested patients.

d Percentage of patients with positive NTM culture. NTM, nontuberculous mycobacteria; *mabs*, M. abscessus subsp. *abscessus*; *mmas*, M. abscessus subsp. *massiliense*; *mbol,* M. abscessus subsp. *bolletii*; MAC, Mycobacterium avium complex.

e Negative patients included those who do not produce sputum or were not investigated for NTM.

### Included isolates and general characteristics.

**FIG 2:**
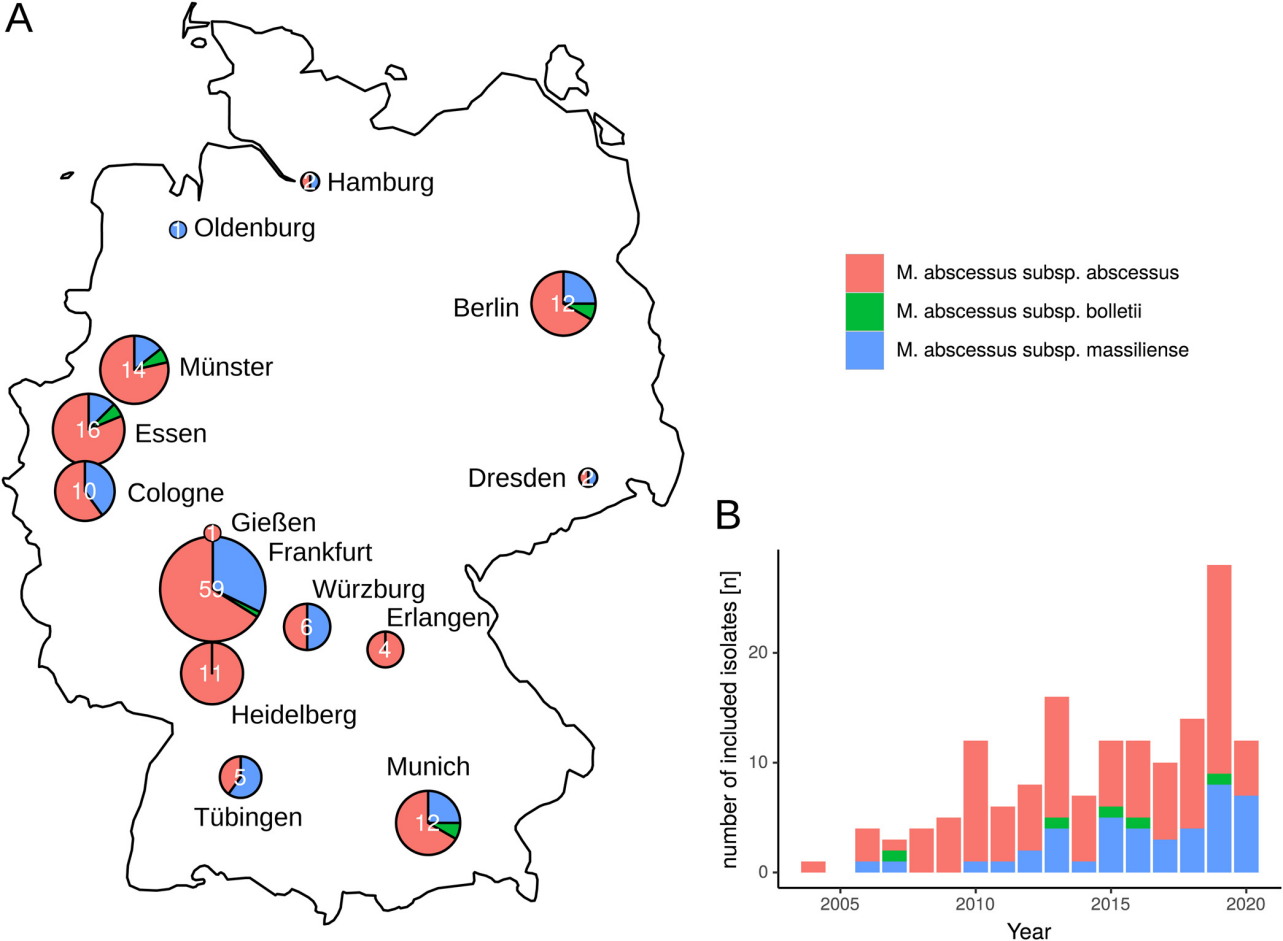
(A) Geographic provenance and subspecies distribution of sequenced CF patient isolates included in this study. (B) Collection date and subspecies distribution of included M. abscessus isolates (*n* = 154).

**FIG 3:**
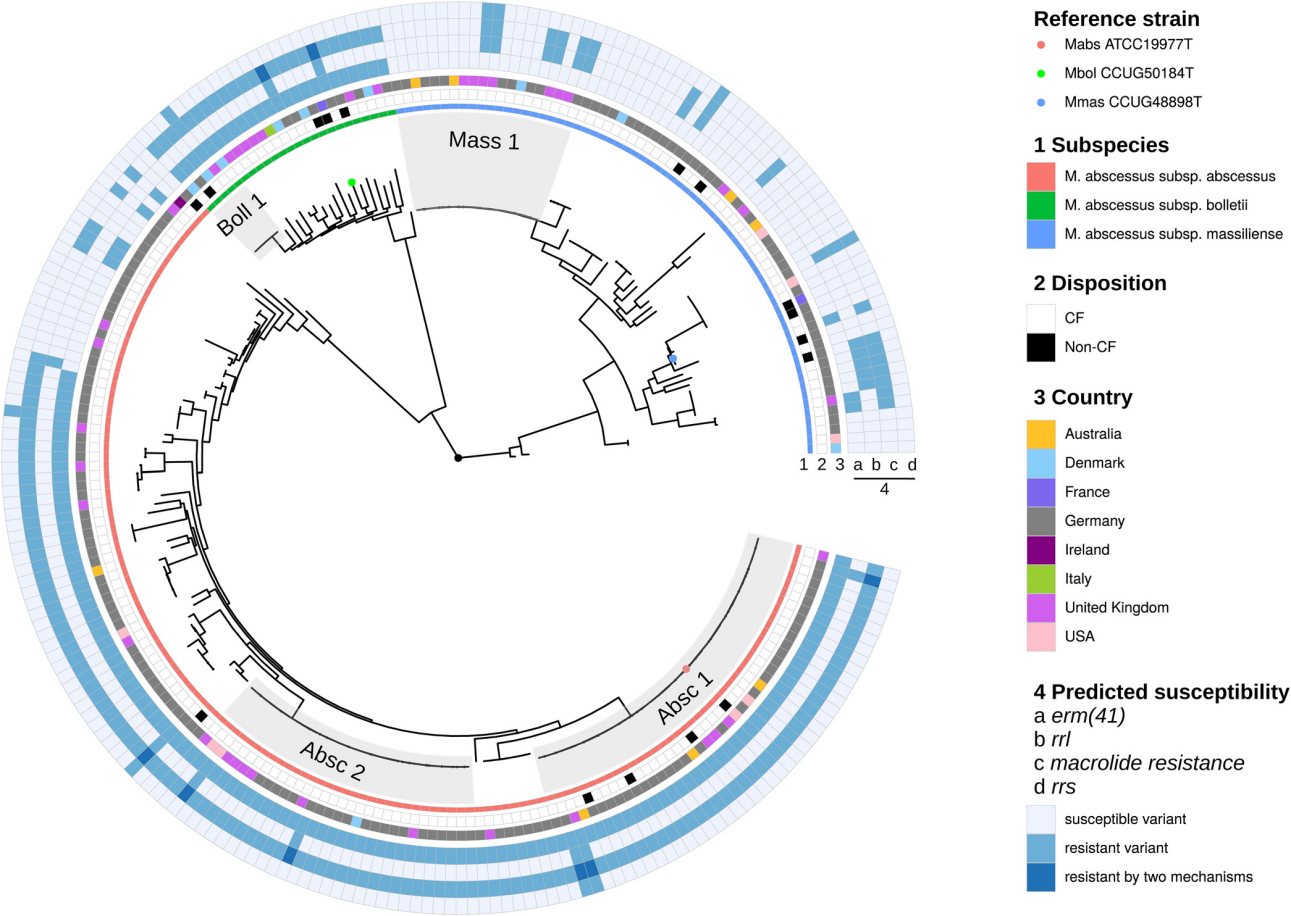
Phylogenetic tree based on whole-genome SNPs in German (*n* = 154) and international (*n* = 55) isolates included in the study. Isolate characteristics (subspecies, CF versus Non-CF, country of origin, and genotypic resistance markers (*erm*(41), *rrl*, *rrs*) are indicated by color code). The reference strains M. abscessus subsp. *abscessus* ATCC 19977, M. abscessus subsp. *massiliense* JCM15300, and M. abscessus subsp. *bolletii* JCM15297 are indicated by circles in the respective colors of the subspecies.

In total, whole-genome data were obtained for 154 M. abscessus isolates from 123 German CF patients in 14 centers (Table 2, Fig. 2). Mean age at first positive culture was 22 years (range 5 to 75 years). Bacterial isolates were initially cultured from sputum (*n* = 104, 84.6%), bronchoalveolar lavage (*n* = 5, 4.1%), bronchial secretion (*n* = 3, 2.4%) and oropharyngeal swabs (*n* = 11, 8.9%). Of the isolates included, 123 were primary isolates and 31 sequential isolates. In primary isolates, subspecies identification by whole-genome sequencing resulted in 83 isolates of subspecies *mabs* (67.5%), 35 isolates of *mmas* (28.5%), and 5 isolates of *mbol* (4.1%) (Table 2, Fig. 3).

**TABLE 2:** Metadata, subspecies, genotypic resistance, and clustering with DCCs of included M. abscessus isolates from German sites

	CF		Non-CF		
Isolate characteristics	Patients [n]	Isolates [n]	Patients [n]	Isolates [n]	*P* value^*a*^
Total	123	154	14	14	
Material					
Sputum	104	129	5	5	**<0.001**
Oropharyngeal swab	11	13	0	0	0.60
Bronchoalveolar lavage	5	9	4	4	**<0.01**
Bronchial secretion	3	3	2	2	0.081
Soft tissue	0	0	3	3	**<0.001**
Isolate type					
Primary isolates	123	123	14	14	
Sequential isolates	17	31	0	0	
Subspecies					
*mabs*	83	107	6	6	0.081
*mmas*	35	42	5	5	0.55
*mbol*	5	5	3	3	**<0.05**
Genotypic antibiotic resistance					
Functional *erm*(41)	78	102	7	7	0.39
*rrl* mutation	15	17	1	1	1
*rrs* mutation	5	5	0	0	1
Clusters					
Absc 1	23	29	3	3	0.73
Absc 2	15	21	0	0	0.36
Mass 1	7	10	0	0	1
Boll 1	0	0	1	1	0.10
Unclustered	30	30	6	6	0.20

a Bold values denote statistical significance with a *P* value < 0.05.

The duration of culture positivity ranged between 0 and 14 years. It was longer in *mabs* (mean 2.68 years, range 0–12 years) and *mmas* (mean 2.93 years, range 0 to 14 years) than in *mbol* (mean 0.8 years, range 0–3 years).

In primary isolates, we could detect 15 isolates (12.2%; 5 *mabs*, 2 *mbol*, 8 *mmas*) that were predicted to be constitutively resistant to macrolides due to mutations in the *rrl* gene, 78 isolates (63.4%, 71 *mabs*, 5 *mbol*, 2 *mmas*) with inducible resistance (functional *erm*(41) gene) and 6 isolates that carried both mechanisms of resistance (4.9%, 4 *mabs* and 2 *mbol*) (Fig. 3). Therefore, 87/123 isolates were genotypically resistant to macrolides (70.7%). Interestingly, 2 *mmas* isolates showed a fully functional *erm*(41) gene. A predicted aminoglycoside resistance mediated by mutations in the *rrs* gene could be detected in 5 isolates (4.1%), resulting in 118 genotypically susceptible isolates in our patient cohort (95.9%).

### Phylogenetic relations.

**FIG 4:**
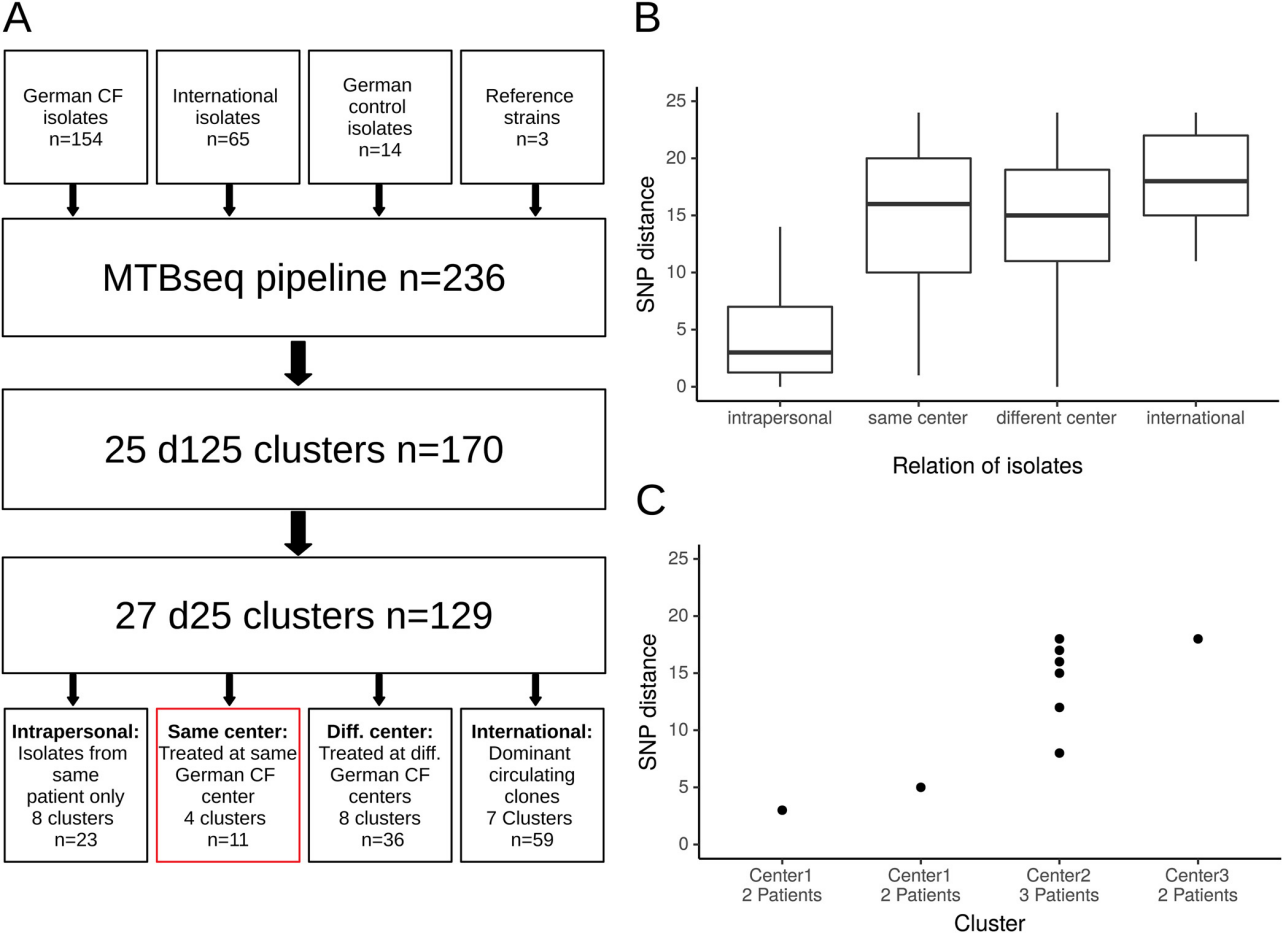
(A) Flowchart of included isolates in cluster analyses (n refers to the respective number of isolates.). (B) SNP distances for four different types of d25 clusters. (C) SNP distances in ‘same center’ clusters, including patients treated at the same German CF center.

SNP distribution for closely related isolates (SNP-distance below 125) showed two distinct peaks: one below 25 SNPs and another below 125 SNPs (Fig. S1), confirming our previously determined thresholds of 25 SNPs (d25) for highly related isolates, and 125 SNPs for closely related isolates (d125) (26). Therefore, we identified 25 d125 clusters with 170 isolates (72.0% of all isolates) and 27 d25 clusters with 129 isolates (54.6% of all isolates) (Fig. 4). Based on a d125 threshold, 37.5% of all German isolates clustered with the DCCs Absc 1, Absc 2 and Mass 1 (*n* = 63, including 3 non-CF isolates). Isolates from CF-patients did not cluster significantly more often with the DCCs than those of non-CF controls (45/123 patients versus 4/14 patients, *P* = 0.378) (Table 2). Isolates from all centers except one (13/14; 92.9%) grouped with the DCCs with a maximum of 125 SNPs distance. However, German isolates did not group with international isolates other than the DCCs with less than 25 SNPs distance.

### Transmission analysis.

Out of the 27 d25 clusters, we could identify 4 clusters that consisted only of patients that have been treated at the same German center (Clusters 1 to 4; Fig. 4C). In center 1, one cluster consisted of two isolates from two twin siblings (*mabs*), suggesting a possible human-to-human transmission or a shared environmental source (Cluster 1). Another cluster at center 1 comprised two isolates from two individuals that were treated at different hospitals and lived in different cities (Cluster 2, *mabs*). Both isolates derived from the same microbiological laboratory that provided diagnostics for the two but distant hospitals (thus originally counted as one center). In addition, these two isolates clustered with international isolates with less than 125 SNPs. In center 2, we identified a *mmas*-cluster comprised of three patients that did not group with international isolates and shared a specific mutation in the *rrs* gene (Cluster 3). However, precise analysis of hospital stays and visits of these three patients could not identify simultaneous hospital visits. In addition, two patients were regularly segregated due to different CF-pathogen status (one colonized with Achromobacter xylosoxidans/one free of Pseudomonas aeruginosa) and the third was a non-CF-patient. Finally, we identified another cluster from a single German center which did not group within the DCCs but with other international isolates (Cluster 4). Unfortunately, more detailed data about these two patients were not available.

## DISCUSSION

This study is the first to provide comprehensive data on colonization and infection with nontuberculous mycobacteria, especially M. abscessus, in CF patients across Germany. We demonstrate that M. abscessus is the most relevant NTM in German CF patients. This is in line with other reports from Europe, whereas in North America MAC is the NTM most frequently isolated among CF patients (10). According to registry data, prevalence of M. abscessus has remained relatively constant between 2015 and 2020 which is in contrast to several previous reports where an increase in prevalence was observed (28, 29). However, in Germany, segregation of NTM-positive CF patients was recommended since 2014 (30). M. abscessus dominates in younger CF patients (including children), whereas the proportion of MAC increases with age. The overall prevalence of NTM of 1.9% to 3.0% in Germany is similar to that reported from other European countries (9). Finally, in more than 50% of all patients NTM culture was not available. Thus, the frequency of M. abscessus may be underestimated. Many patients, particularly younger children, are unable to expectorate and mycobacterial culture might not have been possible (13). Nevertheless, these data clearly indicate that in Germany, the adherence to the CF Foundation/European Cystic Fibrosis Guideline Committee recommendation to annually screen for NTM infection in expectorating patients needs to be improved.

Whole genome sequencing of 154 M. abscessus isolates from 123 CF patients showed that *mabs* is the predominant subspecies in German CF patients (67.5% of all M. abscessus isolates). The dominant circulating clones (Absc 1, Absc 2, and Mass 1) are present in a majority of German CF centers. In total, 37.5% of German isolates grouped within the three dominant DCCs with less than 125 SNPs, but evidence for direct human-to-human transmission is still elusive. On the other hand, we could identify four clusters of highly related isolates that contained only patients that were treated at the same centers in Germany suggesting the possibility of indirect or direct person-to-person transmission. One cluster consisted of two siblings, the second of two patients living in different cities, treated at different hospitals indicating that in these cases in-hospital transmission is not very likely. Two further clusters comprised three and two patients., Although epidemiological analyses did not identify opportunities for direct transmission between the patients in cluster 3, the genetic relatedness and the specific *rrs* mutation suggest that an indirect transmission via an environmental hospital source is possible.

Prior studies from Germany covering only few patients did not find evidence for cross transmission so far (26, 31). However, in CF, the carriage of identical clones, e.g., for P. aeruginosa, and B. cepacia, among siblings is common, suggesting that there might be a household transmission risk for M. abscessus as well. Recent studies have found evidence for cross-infection by M. abscessus (19, 20). Even patients across different centers or even continents may share very closely related isolates (32). Thus, the detection of genotypically related M. abscessus strains during transmission analysis should be interpreted carefully and always with respect to the global molecular epidemiology. It has been hypothesized, that current analytic techniques omitting accessory genes and plasmids might play a role in the seemingly relatedness of some isolates (33). This might be an alternative explanation for the fact that in our study 37.5% of included German isolates cluster with the dominant circulating clones with less than 125 SNPs distance. In addition, d25 clusters that consisted only of ‘one German’ center isolates did not have significant higher SNP distances than those that contained ‘different German center’ isolates. Nevertheless, SNP distances were significantly lower in (sequential) isolates that were only from one patient. On the other hand, our results may also be interpreted with respect to a recent evolutionary model proposed by Bryant et al.: (i) in-host evolution is constrained fitting our observation of highly related intrapersonal isolates with low SNP distances between single isolates, (ii) saltatory evolutional events are supposed to take place in the environment explaining higher SNP distances in interpersonal isolate-to-isolate comparisons within the clusters (21).

Much more isolates were predicted to be resistant to macrolides than to aminoglycosides (70.7% versus 4.1%). Macrolide resistance was mainly mediated by functional *erm*(41) genes in *mabs* and *mbol*. These results are in line with those from global studies, where aminoglycoside resistance remains a rare event (34).

This study has several limitations. First, we could not obtain clinical meta-data for all included patients, as well as information on whether the patients met outside the hospital (for example during private meeting, at patient gatherings etc.). Second, we did not investigate environmental specimens from hospitals to verify if isolates from closely related clusters are present. However, in sporadic and epidemic M. abscessus infections, the pathogen is almost never isolated from the closest environment (35). Third, the control group of non-CF isolates was quite small. However, to the best of our knowledge, this is the largest phylogenomic study on M. abscessus in CF patients in Germany so far.

To conclude, whether the presence of closely related isolates among CF patients has to be regarded as indicator for inter-human transmission remains debatable, especially in the light of current genomic analytic methods. Long read techniques might increase typing resolution in the future by generating closed genomes and by adding accessory genes to improve the discriminatory power of comparative genetic analysis. Nevertheless, we could identify clusters with patients that are infected or colonized with highly related isolates with less than 25 SNPs distance. As health care-associated transmission events cannot be ruled out, hospital infection control practices should be maintained such as segregation of NTM-positive CF patients in order to prevent possible transmission via fomites.

### Conclusion.

The impact of M. abscessus transmission among CF patients varies greatly between different epidemiological studies. In this work, we found no evidence for nosocomial patient-to-patient transmissions, although patients with closely related strains and treated at the same center were identified. As nosocomial transmission could not be completely ruled out, hospital infection control practices for NTM as part of best practice guidelines for the management of CF patients should be maintained. Improved NTM surveillance combined with continuous and systematic epidemiologic investigation of potential transmissions and high-resolution comparative genome analysis techniques will help to guide future evidence-based infection control measures for patients with CF.

## MATERIALS AND METHODS

### Ethical approval.

Ethical consent for this study was given by the ethics committee at University Hospital Frankfurt under file number 20–791. No experiments on animals were involved. As this is a retrospective study, no patient consent was needed.

### German CF registry data.

To assess the overall epidemiological situation in Germany, a database query from the German CF registry was conducted (27, 36). This registry currently includes annual data from over 6,000 CF patients from specialized CF sites, representing approximately 80% of all CF patients in Germany. The database collects demographic, clinical and microbiological data from consenting people with CF since 1995, whereas detailed data for NTM was available since 2015. Thus, for patients with no prior transplant, test results for NTM for the time span of 2015 to 2020 were recorded, as well as testing frequency. NTM-species were divided into M. abscessus, M. avium complex (MAC), others (including M. kansasii, M. fortuitum, M. gordonae, and M. chelonae) and unknown. In addition, patient age at the time of cultivation was recorded. Children were defined as patients with an age <18 years.

### Included isolates.

A representative set of M. abscessus isolates has been collected from 14 German CF centers (located in the cities of Essen, Oldenburg, Cologne, Munich, Berlin, Münster, Dresden, Erlangen, Gießen, Tübingen, Würzburg, Hamburg, Heidelberg, and Frankfurt) for the time period of 2004 to 2020. We recorded the date of cultivation, patient age, type of specimen (sputum, bronchoalveolar lavage, endotracheal swabs) and if isolates were primary or sequential isolates. In addition, the duration between the first and last positive culture was recorded. From Frankfurt University Hospital sequential isolates were included, if available. Bacterial culture was performed on Middlebrook 7H10 agar with oleic albumin dextrose catalase (OADC, Becton, Dickinson, Heidelberg, Germany) at 37°C until visible growth could be detected. M. abscessus species identification was verified by Matrix-assisted-laser desorption ionization-time of flight analysis (MALDI-TOF; Vitek MS; bioMérieux, Nürtingen, Germany) and in case of no identification with the GenoType NTM-DR VER 1.0 (Hain Lifescience, Nehren, Germany). Bacterial cultures were then transferred to the German Reference Center for Mycobacteria at Research Center Borstel for DNA extraction and whole-genome sequencing. Furthermore, 14 M. abscessus isolates from non-CF patients were included as controls.

### DNA extraction and whole-genome sequencing.

We performed cetyltrimethylammonium bromide (CTAB)-chloroform DNA extraction as previously described (37). In brief, cells from solid cultures were dissolved in 400 μL of Tris-EDTA (TE) buffer and then heat inactivated for 20 min at 80°C. Fifty μL of 10-mg/mL lysozyme were added and incubated at 37°C for at least 1 h. Then, proteinase K, sodium dodecyl sulfate, and 5 M NaCl plus CTAB-NaCl were added, each followed by 10 min of incubation at 65°C. Chloroform-isoamyl alcohol was applied for DNA extraction. The supernatant was then transferred into a fresh tube with 450 μL of isopropanol. After an incubation time of at least 30 min at −20°C, the sample was centrifuged and the precipitate was washed with 70% ethanol. Finally, the pellet was dried for 20 min at 60°C and DNA dissolved in 80 μL of TE buffer.

Next-generation sequencing libraries were generated from extracted genomic DNA, using a modified Illumina Nextera library kit protocol (38). Libraries were sequenced in a 2 × 150-bp paired-end run on the Illumina NextSeq 500 instrument (Illumina, San Diego, CA, USA).

### Sequence data analysis.

Macrolide resistance was predicted with the mab-ariba database, including inducible resistance (*erm*(41)) and constitutive resistance (*rrl*) by Lipworth et al. (39). For the detection of aminoglycoside resistance, we added *rrs* gene mutations A1408G, T1406A, and C1409T, as previously described (26).

Sequence reads of all isolates from our study, 65 CF isolates from different countries as described by Bryant et al. (19, 20) (10 from cluster Absc 1, cluster Absc 2, and cluster Mass 1 each, 5 isolates from cluster Boll 1, and 10 unclustered isolates of each subspecies) (Table S1), as well as sequence reads of the reference genomes M. abscessus subsp. *abscessus* ATCC19977, M. abscessus subsp. *bolletii* JCM15297, and M. abscessus subsp. *massiliense* JCM15300, were applied to the MTBseq pipeline with default settings and M. abscessus subsp. *abscessus* ATCC 19977 as a reference genome (GenBank accession NC_010397.1) (40). Thresholds for cluster calling were derived from publications and set at 25 SNPs for highly related isolates and 125 SNPs for closely related ones (19, 24, 26, 41). We calculated a phylogenetic tree from concatenated SNP positions using RaxML v. 8.2.12 with 100 bootstraps and GTR model of nucleotide substitution and the γ mode or rate heterogeneity (42) which was subsequently annotated in *ggtree* (43). The subspecies was derived from clustering with the according reference genomes.

### Transmission analysis and statistical analysis.

A SNP distance below 25 SNPs was considered evocative of a possible recent transmission. Assuming that only patients from the same CF center could directly transmit their bacterial strains nosocomially, we defined putative in-hospital transmission clusters as those in which only patients from the same single German CF center were included. In these clusters, additional epidemiological investigations were performed.

All data were analyzed within the *tidyverse* (44), and graphs were drawn using *ggplot2* (45). Categorical variables are given as numbers and percentages. Statistical tests for categorical variables were performed using the Fisher-Exact-Test. Continuous variables are presented as mean with standard deviation for normally distributed data, and median with interquartile range for nonnormally distributed data. Normality was assessed with the Shapiro-Wilk-Test. The Wilcoxon-Whitney-Mann-Test was used for the detection of statistical differences between groups in nonnormally distributed data. For all statistical tests a significance level of alpha = 0.05 was used.

### Data availability.

All sequence data used in this study was deposited in the European Nucleotide Archive (ENA) under bioproject number PRJEB44160 and can also be accessed via https://ntmscope.github.io/moma_cf_microreact.

## References

[1] Martiniano SL, Nick JA, Daley CL. 2016. Nontuberculous mycobacterial infections in cystic fibrosis. Clin Chest Med 37:83–96. doi:10.1016/j.ccm.2015.11.001. 26857770

[2] Schnabel D, Gaines J, Nguyen DB, Esposito DH, Ridpath A, Yacisin K, Poy JA, Mullins J, Burns R, Lijewski V, McElroy NP, Ahmad N, Harrison C, Parinelli EJ, Beaudoin AL, Posivak-Khouly L, Pritchard S, Jensen BJ, Toney NC, Moulton-Meissner HA, Nyangoma EN, Barry AM, Feldman KA, Blythe D, Perz JF, Morgan OW, Kozarsky P, Brunette GW, Sotir M, Centers for Disease Control and Prevention (CDC). 2014. Notes from the field: rapidly growing nontuberculous *Mycobacterium* wound infections among medical tourists undergoing cosmetic surgeries in the Dominican Republic–multiple states, March 2013–February 2014. MMWR Morb Mortal Wkly Rep 63:201–202. http://www.ncbi.nlm.nih.gov/pubmed/24598597 24598597 PMC4584729

[3] Bonaiti G, Pesci A, Marruchella A, Lapadula G, Gori A, Aliberti S. 2015. Nontuberculous Mycobacteria in Noncystic Fibrosis Bronchiectasis. Biomed Res Int 2015:1–8. doi:10.1155/2015/197950. PMC4461751 26106603

[4] Esther CR, Esserman DA, Gilligan P, Kerr A, Noone PG, Noone PG. 2010. Chronic *Mycobacterium abscessus* infection and lung function decline in cystic fibrosis. J Cyst Fibros 9:117–123. doi:10.1016/j.jcf.2009.12.001. 20071249 PMC3837580

[5] Degiacomi G, Sammartino JC, Chiarelli LR, Riabova O, Makarov V, Pasca MR. 2019. *Mycobacterium abscessus*, an emerging and worrisome pathogen among cystic fibrosis patients. Int J Mol Sci 20:5868. doi:10.3390/IJMS20235868. 31766758 PMC6928860

[6] Raats D, Lorent N, Saegeman V, Vos R, van Ingen J, Verleden G, Van Raemdonck D, Dupont L. 2019. Successful lung transplantation for chronic *Mycobacterium abscessus* infection in advanced cystic fibrosis, a case series. Transpl Infect Dis 21:e13046. doi:10.1111/tid.13046. 30597699

[7] Lobo LJ, Chang LC, Esther CR, Gilligan PH, Tulu Z, Noone PG. 2013. Lung transplant outcomes in cystic fibrosis patients with pre-operative *Mycobacterium abscessus* respiratory infections. Clin Transplant 27:523–529. doi:10.1111/ctr.12140. 23710571

[8] Kavaliunaite E, Harris KA, Aurora P, Dixon G, Shingadia D, Muthialu N, Spencer H. 2020. Outcome according to subspecies following lung transplantation in cystic fibrosis pediatric patients infected with *Mycobacterium abscessus*. Transpl Infect Dis 22. doi:10.1111/tid.13274. 32129923

[9] Zolin A, Orenti A, Naehrlich L, van Jung ARJ. ECFSPR Annual Report 2018. 2020

[10] Adjemian J, Olivier KN, Prevots DR. 2018. Epidemiology of pulmonary nontuberculous mycobacterial sputum positivity in patients with cystic fibrosis in the United States, 2010–2014. Annals ATS 15:817–826. doi:10.1513/AnnalsATS.201709-727OC. PMC6137684 29897781

[11] Kwak N, Dalcolmo MP, Daley CL, Eather G, Gayoso R, Hasegawa N, Jhun BW, Koh W-J, Namkoong H, Park J, Thomson R, van Ingen J, Zweijpfenning SM, Yim J-J. 2019. *Mycobacterium abscessus* pulmonary disease: individual patient data meta-analysis. Eur Respir J 54:1801991. doi:10.1183/13993003.01991-2018. 30880280

[12] Daley CL, Iaccarino JM, Lange C, Cambau E, Wallace RJ, Andrejak C, et al. 2019. Treatment of nontuberculous mycobacterial pulmonary disease: an official ATS/ERS/ESCMID/IDSA clinical practice guideline. 2020. Eur Respir J 56:2000535. doi:10.1183/13993003.00535-2020. PMC8375621 32636299

[13] Floto RA, Olivier KN, Saiman L, Daley CL, Herrmann J-L, Nick JA, Noone PG, Bilton D, Corris P, Gibson RL, Hempstead SE, Koetz K, Sabadosa KA, Sermet-Gaudelus I, Smyth AR, van Ingen J, Wallace RJ, Winthrop KL, Marshall BC, Haworth CS. 2016. US Cystic Fibrosis Foundation and European Cystic Fibrosis Society consensus recommendations for the management of non-tuberculous mycobacteria in individuals with cystic fibrosis: executive summary. Thorax 71:88–90. doi:10.1136/thoraxjnl-2015-207983. 26678435 PMC4717423

[14] Griffith DE, Aksamit T, Brown-Elliott BA, Catanzaro A, Daley C, Gordin F, Holland SM, Horsburgh R, Huitt G, Iademarco MF, Iseman M, Olivier K, Ruoss S, von Reyn CF, Wallace RJ, Winthrop K, Infectious Disease Society of America. 2007. An Official ATS/IDSA Statement: diagnosis, Treatment, and Prevention of Nontuberculous Mycobacterial Diseases. Am J Respir Crit Care Med 175:367–416. doi:10.1164/rccm.200604-571ST. 17277290

[15] Tortoli E, Kohl TA, Brown-Elliott BA, Trovato A, Leão SC, Garcia MJ, Vasireddy S, Turenne CY, Griffith DE, Philley JV, Baldan R, Campana S, Cariani L, Colombo C, Taccetti G, Teri A, Niemann S, Wallace RJ, Cirillo DM. 2016. Emended description of *mycobacterium abscessus mycobacterium abscessus* subsp. Abscessus and *mycobacterium abscessus* subsp. bolletii and designation of *mycobacterium abscessus* subsp. massiliense comb. nov. Int J Syst Evol Microbiol 66:4471–4479. doi:10.1099/ijsem.0.001376. 27499141

[16] Maurer FP, Castelberg C, Quiblier C, Bottger EC, Somoskovi A. 2014. Erm(41)-dependent inducible resistance to azithromycin and clarithromycin in clinical isolates of *Mycobacterium abscessus*. J Antimicrob Chemother 69:1559–1563. doi:10.1093/jac/dku007. 24500188

[17] Maurer FP, Ruegger V, Ritter C, Bloemberg GV, Bottger EC. 2012. Acquisition of clarithromycin resistance mutations in the 23S rRNA gene of *Mycobacterium abscessus* in the presence of inducible erm(41). J Antimicrob Chemother 67:2606–2611. doi:10.1093/jac/dks279. 22833642

[18] Prammananan T, Sander P, Brown BA, Frischkorn K, Onyi GO, Zhang Y, Böttger EC, Wallace RJ. 1998. A single 16S ribosomal RNA substitution is responsible for resistance to amikacin and other 2-deoxystreptamine aminoglycosides in *Mycobacterium abscessus* and *Mycobacterium chelonae*. J Infect Dis 177:1573–1581. doi:10.1086/515328. 9607835

[19] Bryant JM, Grogono DM, Greaves D, Foweraker J, Roddick I, Inns T, Reacher M, Haworth CS, Curran MD, Harris SR, Peacock SJ, Parkhill J, Floto RA. 2013. Whole-genome sequencing to identify transmission of *Mycobacterium abscessus* between patients with cystic fibrosis: a retrospective cohort study. Lancet 381:1551–1560. doi:10.1016/S0140-6736(13)60632-7. 23541540 PMC3664974

[20] Bryant JM, Grogono DM, Rodriguez-Rincon D, Everall I, Brown KP, Moreno P, Verma D, Hill E, Drijkoningen J, Gilligan P, Esther CR, Noone PG, Giddings O, Bell SC, Thomson R, Wainwright CE, Coulter C, Pandey S, Wood ME, Stockwell RE, Ramsay KA, Sherrard LJ, Kidd TJ, Jabbour N, Johnson GR, Knibbs LD, Morawska L, Sly PD, Jones A, Bilton D, Laurenson I, Ruddy M, Bourke S, Bowler IC, Chapman SJ, Clayton A, Cullen M, Daniels T, Dempsey O, Denton M, Desai M, Drew RJ, Edenborough F, Evans J, Folb J, Humphrey H, Isalska B, Jensen-Fangel S, Jönsson B, Jones AM, et al. 2016. Emergence and spread of a human-transmissible multidrug-resistant nontuberculous *mycobacterium*. Science 354:751–757. doi:10.1126/science.aaf8156. 27846606 PMC5142603

[21] Bryant JM, Brown KP, Burbaud S, Everall I, Belardinelli JM, Rodriguez-Rincon D, Grogono DM, Peterson CM, Verma D, Evans IE, Ruis C, Weimann A, Arora D, Malhotra S, Bannerman B, Passemar C, Templeton K, MacGregor G, Jiwa K, Fisher AJ, Blundell TL, Ordway DJ, Jackson M, Parkhill J, Floto RA. 2021. Stepwise pathogenic evolution of *Mycobacterium abscessus*. Science 372:372. doi:10.1126/science.abb8699. PMC7611193 33926925

[22] Ruis C, Bryant JM, Bell SC, Thomson R, Davidson RM, Hasan NA, et al. 2021. Dissemination of *Mycobacterium abscessus* via global transmission networks. Nat Microbiol 6:1279-1288. doi:10.1038/s41564-021-00963-3. PMC8478660 34545208

[23] Redondo N, Mok S, Montgomery L, Flanagan PR, McNamara E, Smyth EG, O'Sullivan N, Schaffer K, Rogers TR, Fitzgibbon MM. 2020. Genomic analysis of *mycobacterium abscessus* complex isolates collected in Ireland between 2006 and 2017. J Clin Microbiol 58. doi:10.1128/JCM.00295-20. PMC7315040 32295892

[24] Tortoli E, Kohl TA, Trovato A, Baldan R, Campana S, Cariani L, Colombo C, Costa D, Cristadoro S, Di Serio MC, Manca A, Pizzamiglio G, Rancoita PM, Rossolini GM, Taccetti G, Teri A, Niemann S, Cirillo DM. 2017. *Mycobacterium abscessus* in patients with cystic fibrosis: low impact of inter-human transmission in Italy. Eur Respir J 50:1602525. doi:10.1183/13993003.02525-2016. 28705942

[25] Lipworth S, Hough N, Weston N, Muller-Pebody B, Phin N, Myers R, et al. 2021. *Mycobacterium abscessus* Genomic Clusters Span Geography and Patient Groups. SSRN Electron J doi:10.2139/ssrn.3745118.

[26] Wetzstein N, Kohl TA, Schultze TG, Andres S, Bellinghausen C, Hügel C, Kempf VAJ, Lehn A, Hogardt M, Serve H, Vehreschild MJGT, Wolf T, Niemann S, Maurer FP, Wichelhaus TA. 2020. Antimicrobial susceptibility and phylogenetic relations in a German cohort infected with *Mycobacterium abscessus*. J Clin Microbiol 58. doi:10.1128/JCM.01813-20. PMC7685876 32938741

[27] Nährlich L, Burkhart M. German Cystic Fibrosis-Registry: Annual Reports 2015–2019. https://www.muko.info/englisch-version/registry.

[28] Qvist T, Gilljam M, Jönsson B, Taylor-Robinson D, Jensen-Fangel S, Wang M, Svahn A, Kötz K, Hansson L, Hollsing A, Hansen CR, Finstad PL, Pressler T, Høiby N, Katzenstein TL. 2015. Epidemiology of nontuberculous mycobacteria among patients with cystic fibrosis in Scandinavia. J Cyst Fibros 14:46–52. doi:10.1016/j.jcf.2014.08.002. 25178871 PMC4298356

[29] RCJOKN. 2019. Nontuberculous Mycobacteria in Cystic Fibrosis. Semin Respir Crit Care Med 40:737–750. doi:10.1055/S-0039-1693706. 31659731

[30] Schwarz C, Hogardt M. 2014. Epidemiologisches Bulletin. Epidemiol Bull. 107. https://www.rki.de/DE/Content/Infekt/Krankenhaushygiene/Kommission/Downloads/Ergaenz_CF.pdf?__blob=publicationFile.

[31] Lewin A, Kamal E, Semmler T, Winter K, Kaiser S, Schäfer H, Mao L, Eschenhagen P, Grehn C, Bender J, Schwarz C. 2021. Genetic diversification of persistent *Mycobacterium abscessus* within cystic fibrosis patients. Virulence 12:2415–2429. doi:10.1080/21505594.2021.1959808. 34546836 PMC8526041

[32] Lipworth S, Hough N, Weston N, Muller-Pebody B, Phin N, Myers R, et al. 2021. Articles Epidemiology of Mycobacterium abscessus in England: an observational study. doi:10.1016/S2666-5247(21)00128-2. PMC8481905 34632432

[33] Davidson RM. 2018. A closer look at the genomic variation of geographically diverse *mycobacterium abscessus* clones that cause human infection and disease. Front Microbiol 9. doi:10.3389/fmicb.2018.02988. PMC6290055 30568642

[34] Bronson RA, Gupta C, Manson AL, Nguyen JA, Bahadirli-Talbott A, Parrish NM, Earl AM, Cohen KA. 2021. Global phylogenomic analyses of *Mycobacterium abscessus* provide context for non cystic fibrosis infections and the evolution of antibiotic resistance. Nat Commun 12:1–10. doi:10.1038/s41467-021-25484-9. 34446725 PMC8390669

[35] Gross JE, Martiniano SL, Nick JA. 2019. Prevention of transmission of *Mycobacterium abscessus* among patients with cystic fibrosis. Curr Opin Pulm Med 25:646–653. doi:10.1097/MCP.0000000000000621. 31436542

[36] Registry | Mukoviszidose e.V. Bundesverband Cystische Fibrose (CF). [cited 30 Sep 2021]. https://www.muko.info/englisch-version/registry/.

[37] de Almeida IN, Carvalho WdS, Rossetti ML, Costa ERD, de Miranda SS. 2013. Evaluation of six different DNA extraction methods for detection of *Mycobacterium tuberculosis* by means of PCR-IS6110: preliminary study. BMC Res Notes 6:561. doi:10.1186/1756-0500-6-561. 24373461 PMC3891981

[38] Baym M, Kryazhimskiy S, Lieberman TD, Chung H, Desai MM, Kishony R. 2015. Inexpensive multiplexed library preparation for megabase-sized genomes. PLoS One 10:e0128036. doi:10.1371/journal.pone.0128036. 26000737 PMC4441430

[39] Lipworth S, Hough N, Buchanan R, Smith EG, Robinson E, Alexander E, Peto T, Crook D, Walker T. 2019. Improved performance predicting clarithromycin resistance in *Mycobacterium abscessus* on an independent dataset. Antimicrob Agents Chemother 63 AAC.00400–19. doi:10.1128/AAC.00400-19. PMC6658746 31160290

[40] Kohl TA, Utpatel C, Schleusener V, De Filippo MR, Beckert P, Cirillo DM, Niemann S. 2018. MTBseq: a comprehensive pipeline for whole genome sequence analysis of *Mycobacterium tuberculosis* complex isolates. PeerJ 6:e5895. doi:10.7717/peerj.5895. 30479891 PMC6238766

[41] Lipworth S, Hough N, Weston N, Muller-Pebody B, Phin N, Myers R, Chapman S, Flight W, Alexander E, Smith EG, Robinson E, Peto TEA, Crook DW, Walker AS, Hopkins S, Eyre DW, Walker TM. 2021. Epidemiology of *Mycobacterium abscessus* in England: an observational study. Lancet Microbe 2:e498. doi:10.1016/S2666-5247(21)00128-2. 34632432 PMC8481905

[42] Stamatakis A. 2014. RAxML version 8: a tool for phylogenetic analysis and post-analysis of large phylogenies. Bioinformatics 30:1312–1313. doi:10.1093/bioinformatics/btu033. 24451623 PMC3998144

[43] Yu G, Smith D, Zhu H, Guan Y, Lam TT-Y. 2017. ggtree: an R package for visualization and annotation of phylogenetic trees with their covariates and other associated data. Methods Ecol Evol 8:28–36. doi:10.1111/2041-210X.12628.

[44] Wickham H, Averick M, Bryan J, Chang W, McGowan L, François R, Grolemund G, Hayes A, Henry L, Hester J, Kuhn M, Pedersen T, Miller E, Bache S, Müller K, Ooms J, Robinson D, Seidel D, Spinu V, Takahashi K, Vaughan D, Wilke C, Woo K, Yutani H. 2019. Welcome to the {tidyverse}. JOSS 4:1686. doi:10.21105/joss.01686.

[45] Wickham H. 2016. ggplot2: elegant graphics for data analysis. Springer-Verlag New York, New York, NY. https://ggplot2.tidyverse.org

