# Supplementary material for: Molecular Epidemiology of Mycobacterium abscessus Isolates Recovered from German Cystic Fibrosis Patients
Source: Microbiol Spectr. 2022 Aug 8;10(4):e01714-22. doi: 10.1128/spectrum.01714-22 (PMC9431180; doi:10.1128/spectrum.01714-22)
Supplement: Supplemental file 1 — Supplemental material. Download spectrum.01714-22-s0001.pdf, PDF file, 0.6 MB [file spectrum.01714-22-s0001.pdf]

Supplementary material:

Table S1: Accession numbers, cluster, year of cultivation and country of isolation of included external isolates that were used for phylogenetic comparison with the German isolates.

Analysis id	Run accession	Sample accession	Cluster	Subspecies	Year	Country
ERZ371284	ERR459789	SAMEA2259579	1abss	mabs	2002	Australia
ERZ371551	ERR343217	SAMEA2068964	1abss	mabs	2009	United Kingdom
ERZ371675	ERR494842	SAMEA2275703	1abss	mabs	2010	United Kingdom
ERZ371789	ERR459826	SAMEA2259620	1absss	mabs	2011	Australia
ERZ371867	ERR340567	SAMEA2071624	1absss	mabs	2012	United Kingdom
ERZ371914	ERR459822	SAMEA2259616	1absss	mabs	2012	Australia
ERZ371993	ERR494937	SAMEA2275808	1absss	mabs	2012	United Kingdom
ERZ372031	ERR484996	SAMEA2275872	1absss	mabs	2013	United Kingdom
ERZ372111	ERR340556	SAMEA2071620	1absss	mabs	2015	United Kingdom
ERZ372266	ERR373959	SAMEA2142635	1absss	mabs	2015	USA
ERZ371238	ERR337803	SAMEA1967286	2absss	mabs	2001	United Kingdom
ERZ371605	ERR343230	SAMEA2068970	2absss	mabs	2010	United Kingdom
ERZ371621	ERR351976	SAMEA2070786	2absss	mabs	2010	United Kingdom
ERZ371868	ERR340568	SAMEA2068740	2absss	mabs	2012	United Kingdom
ERZ371996	ERR494940	SAMEA2275811	2absss	mabs	2012	United Kingdom
ERZ372021	ERR484951	SAMEA2275880	2absss	mabs	2013	United Kingdom
ERZ372038	ERR485003	SAMEA2275881	2absss	mabs	2013	United Kingdom
ERZ372228	ERR369141	SAMEA2149411	2absss	mabs	2015	Denmark
ERZ372262	ERR373955	SAMEA2148059	2absss	mabs	2015	USA
ERZ372329	ERR374019	SAMEA2150987	2absss	mabs	2015	USA
ERZ371269	ERR459771	SAMEA2259560	1mass	mmas	2001	Australia
ERZ371379	ERR337839	SAMEA2070653	1mass	mmas	2005	United Kingdom
ERZ371422	ERR337869	SAMEA2070662	1mass	mmas	2006	United Kingdom
ERZ371592	ERR490687	SAMEA2275670	1mass	mmas	2009	United Kingdom
ERZ371697	ERR340517	SAMEA2071556	1mass	mmas	2011	United Kingdom
ERZ372013	ERR459928	SAMEA2259648	1mass	mmas	2013	Australia
ERZ371806	ERR473276	SAMEA2275636	1mass	mmas	2011	United Kingdom
ERZ372231	ERR369144	SAMEA2163411	1mass	mmas	2015	Denmark
ERZ372253	ERR337820	SAMEA2071357	1mass	mmas	2015	United Kingdom
ERZ372365	ERR473269	SAMEA2275629	1mass	mmas	2015	United Kingdom
ERZ371470	ERR459959	SAMEA2259686	unclustered	mabs	2007	Australia
ERZ371661	ERR490697	SAMEA2275687	unclustered	mabs	2010	United Kingdom
ERZ371791	ERR459832	SAMEA2259631	unclustered	mmas	2011	Australia
ERZ371895	ERR337770	SAMEA2071240	unclustered	mmas	2012	United Kingdom
ERZ372018	ERR484948	SAMEA2275870	unclustered	mabs	2013	United Kingdom
ERZ372160	ERR330835	SAMEA2071201	unclustered	mabs	2015	Ireland
ERZ372248	ERR330874	SAMEA2070535	unclustered	mabs	2015	United Kingdom
ERZ372295	ERR373986	SAMEA2156366	unclustered	mabs	2015	USA
ERZ371240	ERR337811	SAMEA2070829	unclustered	mabs	2001	United Kingdom
ERZ371441	ERR459926	SAMEA2259645	unclustered	mmas	2006	Australia
ERZ371714	ERR340496	SAMEA2071549	unclustered	mabs	2011	United Kingdom
ERZ371875	ERR330781	SAMEA2071184	unclustered	mabs	2012	United Kingdom
ERZ372042	ERR494979	SAMEA2275869	unclustered	mabs	2013	United Kingdom

ERZ372205	ERR363442	SAMEA2150176	unclustered	mmas	2015	Denmark
ERZ372288	ERR373979	SAMEA2155514	unclustered	mmas	2015	USA
ERZ372294	ERR373985	SAMEA2145943	unclustered	mmas	2015	USA
ERZ372257	ERR373942	SAMEA2143632	unclustered	mmas	2015	Denmark
ERZ372157	ERR330756	SAMEA2070588	unclustered	mmas	2015	United Kingdom
ERZ372128	ERR349279	SAMEA2156007	unclustered	mmas	2015	USA
ERZ372029	ERR484993	SAMEA2275866	unclustered	mmas	2013	United Kingdom
ERZ371837	ERR494869	SAMEA2275734	1boll	mbol	2011	United Kingdom
ERZ372214	ERR363457	SAMEA2150262	1boll	mbol	2015	Denmark
ERZ372223	ERR369136	SAMEA2147405	1boll	mbol	2015	Denmark
ERZ372236	ERR369149	SAMEA1971359	1boll	mbol	2015	Denmark
IF2*	NA	NA	1boll	mbol	2003	Italy
ERZ371667	ERR490703	SAMEA2275694	unclustered	mbol	2010	United Kingdom
ERZ371704	ERR340523	SAMEA2071558	unclustered	mbol	2011	United Kingdom
ERZ371737	ERR330787	SAMEA2071186	unclustered	mbol	2011	United Kingdom
ERZ371881	ERR337753	SAMEA2070302	unclustered	mbol	2012	United Kingdom
ERZ371967	ERR494914	SAMEA2275782	unclustered	mbol	2012	United Kingdom
ERZ372010	ERR363451	SAMEA1966577	unclustered	mbol	2013	United Kingdom
ERZ372100	ERR340545	SAMEA2070907	unclustered	mbol	2015	United Kingdom
ERZ372212	ERR363455	SAMEA2160080	unclustered	mbol	2015	Denmark
ERZ372221	ERR369134	SAMEA2153565	unclustered	mbol	2015	Denmark
ERZ372224	ERR369137	SAMEA2149450	unclustered	mbol	2015	Denmark

mabs – *M. abscessus* subsp. *abscessus*; mmas – *M. abscessus* subsp. *massiliense*; mbol – *M. abscessus* subsp. *bolletii*.
*From internal database at research center Borstel. Tortoli et al. Mycobacterium abscessus in patients with cystic fibrosis: Low impact of inter-human transmission in Italy. European Respiratory Journal. European Respiratory Society. https://doi.org/10.1183/13993003.02525-2016

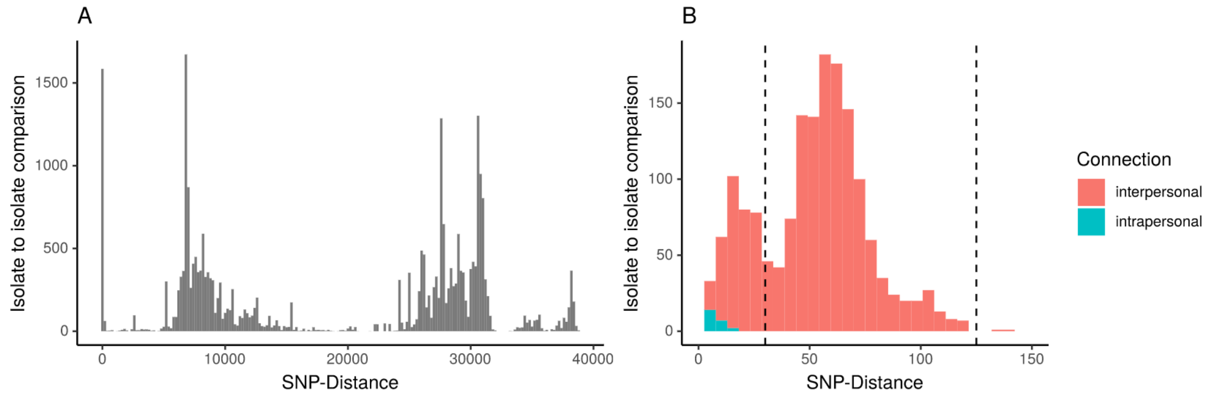

Figure S1: SNP-distance distribution for all isolate-to-isolate comparisons in this study (A) and zoom-in for closely related isolates (B). Vertical dashed lines mark our thresholds of 25 and 125 SNPs.
